# Stabilization of High‐Pressure Phase of Face‐Centered Cubic Lutetium Trihydride at Ambient Conditions

**DOI:** 10.1002/advs.202401642

**Published:** 2024-05-22

**Authors:** Xin Li, Ying Wang, Yuhao Fu, Simon A. T. Redfern, Shuqing Jiang, Pinwen Zhu, Tian Cui

**Affiliations:** ^1^ Synergetic Extreme Condition High‐Pressure Science Center State Key Laboratory of Superhard Materials College of Physics Jilin University Changchun 130012 China; ^2^ Asian School of the Environment Nanyang Technological University 50 Nanyang Avenue Singapore 639798 Singapore; ^3^ Institute of High Pressure Physics School of Physical Scientific and Technology Ningbo University Ningbo 315211 China

**Keywords:** high pressure chemistry, lutetium hydride, materials science, superconductivity

## Abstract

Superconductivity at room temperature and near‐ambient pressures is a highly sought‐after phenomenon in physics and materials science. A recent study reported the presence of this phenomenon in N‐doped lutetium hydride [*Nature* 615, 244 (2023)], however, subsequent experimental and theoretical investigations have yielded inconsistent results. This study undertakes a systematic examination of synthesis methods involving high temperatures and pressures, leading to insights into the impact of the reaction path on the products and the construction of a phase diagram for lutetium hydrides. Notably, the high‐pressure phase of face‐centered cubic LuH_3_ (fcc‐LuH_3_) is maintained to ambient conditions through a high‐temperature and high‐pressure method. Based on temperature and anharmonic effects corrections, the lattice dynamic calculations demonstrate the stability of fcc‐LuH_3_ at ambient conditions. However, no superconductivity is observed above 2 K in resistance and magnetization measurements in fcc‐LuH_3_ at ambient pressure. This work establishes a comprehensive synthesis approach for lutetium hydrides, thereby enhancing the understanding of the high‐temperature and high‐pressure method employed in hydrides with superconductivity deeply.

## Graphical abstract

Absence of superconductivity in face‐centered cubic LuH_3_, which is maintained to ambient conditions through a high‐temperature and high‐pressure method using large volume press.

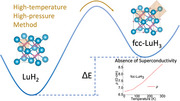

## 1 Introduction

Over the past decade, there has been a burgeoning exploration into hydride‐based superconductors with high critical temperature (*T*_c_).^[^ ^1^, ^2^, ^3^, ^4^, ^5^, ^6^, ^7^, ^8^ ^]^ Two notable experimental achievements involve H_3_S with a high *T*_c_ of 203 K,^[^ ^9^ ^]^ and LaH_10_ with a high *T*_c_ of 250 K.^[^ ^10^ ^]^ These milestones provide significant confidence in the pursuit of hydride‐based superconductors at room temperature. A recent study reported the occurrence of superconductivity at room temperature and near‐ambient pressures of 294 K and 1 GPa in N‐doped lutetium hydride.^[^ ^11^ ^]^ Subsequent to this report, a significant amount of experimental and theoretical work has been undertaken to reproduce and understand this superconducting phenomenon.^[^ ^12^, ^13^, ^14^, ^15^, ^16^, ^17^, ^18^, ^19^, ^20^, ^21^, ^22^, ^23^, ^24^, ^25^, ^26^, ^27^, ^28^, ^29^, ^30^, ^31^, ^32^, ^33^, ^34^, ^35^, ^36^, ^37^ ^]^ However, these efforts failed to observe the phenomenon of superconductivity in N‐doped lutetium hydrides, leading to the retraction of the paper.^[^ ^38^ ^]^ Several experimental studies have reported the absence of near‐ambient superconductivity in N‐doped lutetium hydride under various conditions. For instance, Ming et al. observed the absence of near‐ambient superconductivity in N‐doped lutetium hydride under pressure of 40.1 GPa through magnetic and electric measurements.^[^ ^12^ ^]^ Similarly, Xing et al. found no superconducting transition at temperatures ranging from 1.8 to 300 K and pressures from 0 to 38 GPa.^[^ ^13^ ^]^ Theoretical works contended that there is no stable ternary structure in the Lu–H–N chemical system at low‐pressure conditions and proposed some Lu–H–N candidates, including Lu_8_H_21_N,^[^ ^14^ ^]^ Lu_2_H_5_N,^[^ ^15^ ^]^ and Lu_20_H_2_N_17_.^[^ ^16^ ^]^ Despite extensive structure predictions by other groups, no explanations have been provided for the observed superconductivity near ambient conditions.^[^ ^17^, ^18^, ^19^, ^20^ ^]^

Multiple lines of evidence, including sample color, X‐ray diffraction data, and thermal and dynamical stability, collectively support the assertion that the reported N‐doped Lutetium hydride corresponds to N‐doped LuH_2_ (LuH_2±x_N_y_), as opposed to N‐doped LuH_3_ (LuH_3±x_N_y_). The lattice parameter of cubic N‐doped lutetium hydride was determined to be 5.0289,^[^ ^11^ ^]^ 5.032,^[^ ^12^ ^]^ and 5.040 Å,^[^ ^13^ ^]^ respectively, closely aligning with the lattice parameter of 5.033 Å for LuH_2_. The observed blue coloration of the samples is consistent with the characteristic color of LuH_2_.^[^ ^11^, ^12^, ^13^, ^26^ ^]^ Enthalpy calculations reveal that LuH_2_ represents the ground state structure, whereas LuH_3_ assumes a metastable structure.^[^ ^14^, ^16^, ^17^, ^19^ ^]^ Furthermore, phonon spectrum calculations indicate that LuH_2_ is dynamically stable, in contrast to the instability exhibited by LuH_3_.^[^ ^11^, ^19^ ^]^

Two structural forms of LuH_3_ are identified: a hexagonal structure with $P\bar{3}c1$ space group (hcp‐LuH_3_) and a face‐centered cubic structure with *Fm* $\bar{3}$ *m* space group (fcc‐LuH_3_). Hcp‐LuH_3_ was found to be stable at ambient conditions and transformed to fcc‐LuH_3_ at 12 GPa. Fcc‐LuH_3_, in turn, transformed back to hcp‐LuH_3_ upon releasing the pressure to ambient conditions.^[^ ^39^, ^40^, ^41^ ^]^ The reported superconductivity of fcc‐LuH_3_ occurred at ≈12.4 K at 122 GPa.^[^ ^42^ ^]^

In this work, we systematically prepared pure bulk samples of lutetium hydrides, including LuH_2_ and hcp‐LuH_3_, and notably managed to maintain fcc‐LuH_3_ to ambient conditions, using high‐temperature and high‐pressure method with large volume press (LVP) apparatus. We investigated the effect of the reaction pathway on the products and developed a preparation phase diagram. The fcc‐LuH_3_ exhibits obvious distinctions from LuH_2_, specifically, the lattice parameter for fcc‐LuH_3_ is 5.156(1) Å, larger than 5.033 Å observed for LuH_2_. Additionally, fcc‐LuH_3_ is characterized by a dark black coloration, in contrast to the blue color associated with LuH_2_. Density functional theory (DFT) calculations, including anharmonic and temperature effects, confirm the stability of fcc‐LuH_3_ under ambient conditions. The hydrogen content of fcc‐LuH_3_ is confirmed using temperature‐programmed desorption (TPD) and Nuclear Magnetic Resonance (NMR) methods. Importantly, our low‐temperature resistance and magnetization measurements confirmed the absence of a superconducting transition in the fcc‐LuH_3_ phase.

## 2 Results and Discussion

### 2.1 Synthesis of fcc‐LuH_3_

**Figure 1:**
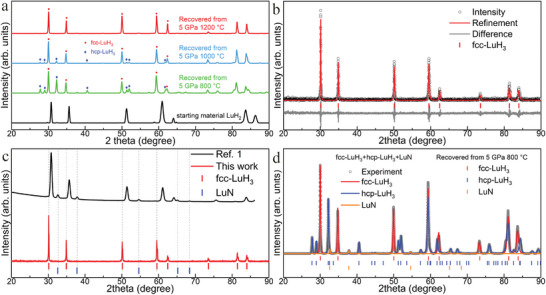
XRD patterns of the synthesized lutetium hydride samples. All XRD patterns are obtained utilizing Cu Kα radiation (λ = 1.5406 Å) at ambient conditions. a) XRD patterns corresponding to various synthetic conditions. The phases of fcc‐LuH_3_ and hcp‐LuH_3_ are indicated by pink dots and blue diamonds, respectively. b) Experimental XRD pattern and Rietveld refinement of fcc‐LuH_3_. The experiment data, refinement result, and residues are denoted in black circles, pink line, and gray line, respectively. The expected positions of diffraction peaks for fcc‐LuH_3_ are indicated by pink ticks. c) Comparison between reported LuH_3±x_N_y_^[^ ^11^ ^]^ and fcc‐LuH_3_. d) Indexing of sample and identification of impurities. The gray circles, pink line, blue line and orange line represent the experimental XRD pattern, simulated XRD of fcc‐LuH_3_, simulated XRD of hcp‐LuH_3_, and LuN, respectively. The expected positions of diffraction peaks for fcc‐LuH_3_, hcp‐LuH_3_, and LuN are marked by pink, blue, and orange.

The synthesis of the fcc‐LuH_3_ was investigated under extreme conditions of high temperature and pressure. LuH_2_ was chosen as the starting material, with BH_3_NH_3_ serving as the solid hydrogen source. The fcc‐LuH_3_ was synthesized at a high pressure of 5 GPa and temperatures exceeding 800 °C. A dual‐phase composition of fcc‐LuH_3_ and hcp‐LuH_3_ was observed after recovery to ambient conditions from 5 GPa and 800 °C (**Figure** **1**a). As the temperature increases to 1000 °C, the hcp‐LuH_3_ phase diminishes significantly, transforming into fcc‐LuH_3_. Pure fcc‐LuH_3_ was obtained after recovery to ambient conditions from the conditions of 5 GPa and 1200 °C. Figure 1b presents a typically refined X‐ray diffraction (XRD) pattern of the face‐centered cubic structure. To confirm the reliability of the synthesis conditions, we conducted 10 sets of synthesis experiments. The XRD patterns indicate that the synthesis conditions of 5 GPa and 1100 °C (1200 °C) for 1 h are dependable for synthesizing pure fcc‐LuH_3_ (Figure S1, Supporting Information). We compared the XRD pattern of the fcc‐LuH_3_ produced in this study with the reported LuH_3±x_N_y_,^[^ ^11^ ^]^ which is actually LuH_2_. Each diffraction peak of fcc‐LuH_3_ exhibits a leftward shift in comparison to LuH_2_, indicating that the diffraction peaks of fcc‐LuH_3_ have a larger *d*‐spacing, *i*.*e*., fcc‐LuH_3_ has a larger lattice parameter (Figure 1c). The refined lattice parameter of fcc‐LuH_3_ is a = 5.156(1) Å, which is noticeably larger than a = 5.033 Å of LuH_2_. An earlier study indicates that the lattice parameter of fcc‐LuH_3_ extrapolated to ambient conditions through high‐pressure volume is 5.12(2) Å,^[^ ^40^ ^]^ a value closely resembling the 5.156(1) Å found in this study. Moreover, XRD exhibits limited sensitivity to hydrogen occupation, potentially allowing for dislocations within the material leading to non‐stoichiometric composition, *i*.*e*., LuH_3‐x_, while still showing the overall fcc symmetry. The discussion about hydrogen occupations will be presented in the subsequent section.

In previous works, yttrium hydrides present comparable results, with two distinct compounds of fcc‐YH_3_ and hcp‐YH_3_. The fcc‐YH_3_ is synthesized through a transformation to the hcp‐YH_3_ structure under conditions of high temperature and pressure, and it can be quenched to ambient conditions.^[^ ^43^, ^44^ ^]^ Despite the instability of the fcc‐YH_3_ at ambient pressure, it has been noted that high‐temperature and high‐pressure conditions play a crucial role in maintaining the presence of high‐pressure phase fcc‐YH_3_ at ambient pressure.

### 2.2 Theoretically Thermal and Dynamical Stability of fcc‐LuH_3_

**Figure 2:**
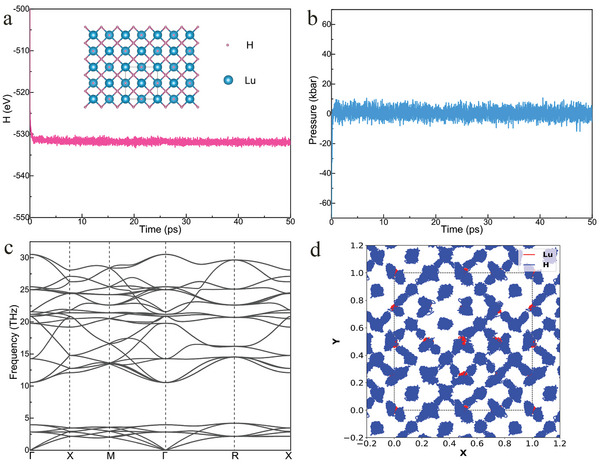
The thermal and dynamic stability of fcc‐LuH_3_ at ambient conditions. a) Convex hull of Lu‐H system at 300 K. b) Energy minimization for molecular dynamics simulation. The inset image is the crystal structure after MD simulation. c) Anharmonic phonon dispersions of fcc‐LuH_3_ at 0 GPa and 300 K. d) AIMD trajectories of fcc‐LuH_3_ at 0 GPa and 300 K. H‐ions are represented with small green spheres, and Lu ions are shown with red spheres.

Theoretical calculations were performed on the fcc‐LuH_3_ compound, utilizing density functional theory (DFT). At 0 K, both LuH_2_ and hcp‐LuH_3_ exhibit thermal stability, as indicated by their positions on the convex hull. However, fcc‐LuH_3_ is situated above the convex hull, with an enthalpy of 86 eV atom^−1^ higher than that of ground state hcp‐LuH_3_, indicating fcc‐LuH_3_ is metastable (Figure S2). The calculated phonon dispersion of fcc‐LuH_3_ shows severe imaginary frequencies across the entire Brillouin zone at 0 K. These were obtained via the real‐space supercell approach, considering solely interatomic harmonic interactions. The presence of imaginary frequencies implies lattice instability (Figure S3, Supporting Information), which is consistent with previous theoretical works.^[^ ^11^, ^14^, ^45^ ^]^ However, it is important to note that anharmonicity corrections are particularly significant for hydrides, *i*.*e*., the zero‐point vibration energy of hydrogen atoms can even modify the ground state structure. The interatomic anharmonic interactions may play a substantial role and likely need to be taken into account at finite temperatures. We used temperature‐dependent effective potential (TDEP) to obtain finite‐temperature renormalized phonon spectra by extraction ab initio molecular dynamics (AIMD) data. We conducted an AIMD simulation of fcc‐LuH_3_ using 128‐atom supercells at 300 K with experimental lattice parameter (a = 5.156 Å). No evidence of phase transition was observed during 60 ps AIMD simulations (**Figure** **2**a). We found that the system pressure fluctuated slightly at ≈0 GPa throughout the simulation (Figure 2b), implying the reliability of the AIMD simulation. Most importantly, no imaginary frequencies were observed in the phonon dispersion at 300 K (Figure 2c), demonstrating the dynamic stability of fcc‐LuH_3_ at ambient conditions. At a low temperature of 50 K, the structure of fcc‐LuH_3_ also maintains dynamic stability (Figure S4, Supporting Information). Figure 2d depicts the trajectories of fcc‐LuH_3_, indicating vigorous movement of hydrogen atoms over a large area. A systematic study on the temperature and anharmonic lattice effects on lutetium trihydride is noted in the literature.^[^ ^45^ ^]^ Finite temperatures are found to be imperative for stabilizing the fcc‐LuH_3_ phase, with dynamic stability observed above 200 K. Considering temperature, there is a significant expansion in lattice parameter. Our calculations using TDEP align with results obtained through stochastic self‐consistent harmonic approximation (SSCHA). Owing to the weak X‐ray scattering cross section of H, XRD does not provide constraints on the crystallographic position of H atoms. The H positions are also determined by geometry optimization of DFT calculation. Two types of hydrogen atoms are identified in fcc‐LuH_3_: octahedral hydrogen (H_O_) atoms located at the center of Lu octahedra, with Wyckoff position 4b (1/2,1/2,1/2), and tetrahedral hydrogen (H_T_) atoms positioned at the center of Lu tetrahedra, with Wyckoff position 8c (1/4,1/4,1/4).

### 2.3 Synthesis of LuH_2_

The synthesis of LuH_2_ has been the subject of considerable investigation in recent studies.^[^ ^12^, ^13^ ^]^ In an effort to explore the formation of LuH_2_ under high‐pressure conditions, we conducted high‐pressure experiments at extreme temperatures and pressures utilizing LVP. Lu pieces were selected as starting materials, and mixed powder of NH_4_Cl:CaH_2_ (2:8) served as the solid hydrogen source. The synthesis of LuH_2_ was accomplished under conditions of 3 GPa and 500 °C for a duration of 5 h. The XRD diffraction pattern shows a typical fcc structure of LuH_2_ (Figure S5, Supporting Information). The refinement parameter, determined *a* = 5.036 (1) Å, was found to be in accordance with recently reported results for LuH_3±x_N_y_^[^ ^11^ ^]^ and LuH_2±x_N_y_.^[^ ^12^, ^13^ ^]^

### 2.4 Synthesis of hcp‐LuH_3_

Both LuH_2_ and hcp‐LuH_3_ are stable on the convex hull. However, LuH_2_ is the preferred structure due to insufficient hydrogen. It is hypothesized that the mixture powder of NH_4_Cl:CaH_2_ (2:8) cannot provide enough hydrogen for this combination reaction, and CaH_2_ may not undergo a replacement reaction with lutetium. A dual‐phase of fcc‐LuH_3_ and hcp‐LuH_3_ is synthesized at 5 GPa and 800 °C for 1 h, using LuH_2_ as the starting material and NH_3_BH_3_ as the internal hydrogen source. The hcp‐LuH_3_ gradually transforms to fcc‐LuH_3_ from 800 to 1200 °C at 5 GPa (Figure 1a). To synthesize pure hcp‐LuH_3_, Lu pieces were selected as the starting material and NH_3_BH_3_ as the internal hydrogen source, given its superior ability to decompose hydrogen. The pure hcp‐LuH_3_ is synthesized under extreme conditions of 5 GPa and 1100 °C for 1 h. Figure S6 (Supporting Information) displays a typical XRD diffraction pattern of the hcp structure. The refinement parameters are a = 6.182(1) Å and c = 6.441(1) Å.

### 2.5 Elimination of Impurities of Lu and Lu_2_O_3_

Considering potential impurities, we have ruled out the presence of Lu and Lu_2_O_3_ with careful consideration (Figure 1d; Figure S7, Supporting Information). The lack of LuH_2_ diffraction peaks indicates that all LuH_2_ react with excess hydrogen and form LuH_3_. The hexagonal phase of Lu metal is known to be stable up to at least 40 GPa.^[^ ^46^ ^]^ Our XRD pattern reveals the absence of Lu peaks, indicating that excess hydrogen facilitated the complete reaction of Lu with hydrogen, forming lutetium hydrides. On the other hand, the cubic phase of Lu_2_O_3_ remains stable up to 12 GPa, undergoing a transformation to a monoclinic phase at higher pressures.^[^ ^47^, ^48^ ^]^ In our observations, the monoclinic phase of Lu_2_O_3_ was not detected, thus ruling out its presence. Although the main peak positions of cubic Lu_2_O_3_ closely resemble those of fcc‐LuH_3_, there exists a discernible difference between them, ≈0.2°. The larger lattice parameter of Lu_2_O_3_ (a = 8.4 Å) results in additional diffraction peaks, such as those at 23° and 45°. The absence of these peaks further substantiates the absence of Lu_2_O_3_ in the sample. We also examined the purity of the starting materials Lu and LuH_2_, and there are only tiny traces of impurities (Figures S8 and S9, Supporting Information). Because the XRD peaks are too weak, the impurities could not be identified. Here we assume that the impurities might be lutetium oxides or hydroxides since our synthesized fcc‐LuH_3_ is pure and it's probable that these impurities will be reduced by hydrogen and transformed into hydrides through reactions.

### 2.6 Comparison of Colors, Crystal Structure, and Lattice Parameters between Lu and Lutetium Hydrides

**Figure 3:**
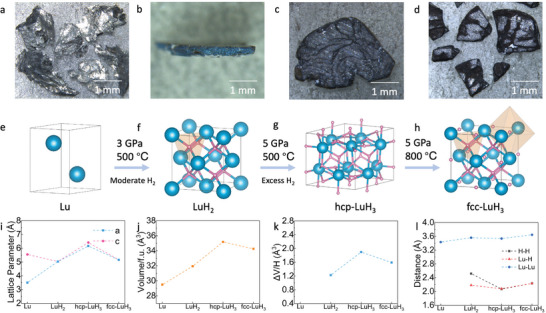
Micrographs, crystal structures, and lattice parameters of lutetium hydrides. a–d) Optical photos of reactant Lu metal synthesized LuH_2_, synthesized hcp‐LuH_3_, and synthesized fcc‐LuH_3_. e–h) Schematic representation of the different experimental runs, synthesized conditions, and crystal structures of Lu, LuH_2_, hcp‐LuH_3_, and fcc‐LuH_3_. i–l) Lattice parameters, unit cell volume, volume expansion ΔV, and nearest‐neighbor distances for H–H, Lu–H, and Lu–Lu in Lu, LuH_2_, hcp‐LuH_3_, and fcc‐LuH_3_.

Commercially available Lu pieces and LuH_2_ powder were used as starting materials. The Lu pieces exhibit a metallic silver color (**Figure** **3**a), while LuH_2_ synthesized under high‐temperature and high‐pressure conditions displays a bright blue color (Figure 3b). Both hcp‐LuH_3_ and fcc‐LuH_3_ are black and dark black respectively, each with a metallic sheen (Figure 3c,d). Lu possesses a hexagonal crystal structure with a *P*6_3_/*mmc* space group (Figure 3e). LuH_2_ has a fcc structure with an $Fm\bar{3}m$ space group, with H atoms occupying the interstices of Lu sublattice tetrahedrons (Figure 3f). Hcp‐LuH_3_ has a hexagonal structure with a $P\bar{3}c1$ space group (Figure 3g). Fcc‐LuH_3_ has an fcc structure with $Fm\bar{3}m$ space group, H atoms occupy the interstices of Lu sublattice tetrahedrons and octahedrons. The phase transition sequence of high‐temperature and high‐pressure synthesis is depicted in Figure 3e–h. Lattice parameters and unit cell volume are presented in Figure 3i,j, illustrating a gradual increase in the volume of unit cells from Lu to LuH_2_ to hcp‐LuH_3_ due to hydrogen absorption. However, there is a 2.61% collapse in the unit cell volume from 35.19(2) Å^3^ f.u^−1^. for hcp‐LuH_3_ to 34.27(2) Å^3^ f.u.^−1^ for fcc‐LuH_3_. Figure 3k visually represents the hydrogen‐induced volume expansion, with ΔV/H values of 1.23 Å^3^ f.u.^−1^ for LuH_2_ and 1.90 Å^3^/f.u. for hcp‐LuH_3_. Due to the volume collapse from hcp‐LuH_3_ to fcc‐LuH_3_, the volume expansion ΔV/H is reduced to 1.59 Å^3^/f.u. Figure 3l illustrates the nearest‐neighbor H–H, Lu–H, and Lu–Lu distances. The closest Lu‐Lu distances progressively increase from 3.561(1) Å in LuH_2_ to 3.640(1) Å in fcc‐LuH_3_. The nearest‐neighbor Lu–H distance expands from 2.181(1) Å in LuH_2_ to 2.233(1) Å in fcc‐LuH_3_, while the nearest‐neighbor H–H distance decreases from 2.518(1) Å in LuH_2_ to 2.233(1) Å in fcc‐LuH_3._

### 2.7 Determination of Nitrogen Doping in fcc‐LuH_3_

**Figure 4:**
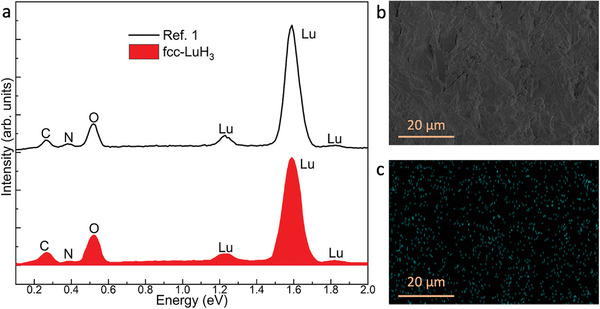
Elemental composition of fcc‐LuH_3_ at ambient conditions. a) EDS spectra of synthesized fcc‐LuH_3_. b,c) SEM image and EDS mapping image for N element at same sample surface area of LuH_3_.

The N‐doped LuH_3_±xNy reported by Dasenbrock‐Gammon et al. is identified as a dual‐phase of LuH_2_ and LuN, based on the XRD patterns and the color of the sample. In our study, we also detected the LuN phase in the fcc‐LuH_3_ sample when only the surface of the sample was cleaned (Figure S10, Supporting Information). The LuN, with a lattice parameter of a = 4.753(1) Å, aligns with the recent work.^[^ ^11^ ^]^ It forms readily on the surface of the fcc‐LuH_3_ sample due to the decomposition of NH_3_BH_3_. Upon meticulous abrasion and cleaning of the sample surface, we successfully obtained pure fcc‐LuH_3_ samples (Figure 1b; Figure S1, Supporting Information). Energy‐dispersive X‐ray spectroscopy (EDS) was performed on fcc‐LuH_3_ samples. The EDS spectrum is in agreement with the reported LuH_3±x_N_y_ and shows a weak peak from nitrogen and indicates a slight nitrogen doping in fcc‐LuH_3_ (**Figure** **4**a). Given the facile formation of LuN, we attribute this doping phenomenon to the presence of LuN. Figure 4b,c shows a scanning electron microscope (SEM) image and EDS mapping of a cross‐section of fcc‐LuH_3_, respectively. The EDS mapping reveals the spatial distribution of nitrogen in our fcc‐LuH_3_ sample and illustrates that nitrogen is widespread homogeneously in the sample with an average nitrogen composition of 1.3 wt.%. Despite theoretical predictions of various metastable Lu–N–H compounds, such as Lu_8_H_21_N and Lu_2_H_5_N, with lattice parameters consistent with LuH_2_,^[^ ^15^, ^19^, ^49^ ^]^ quantifying the N content and doping structure in our investigation is a challenge.

### 2.8 The Hydrogen Content of fcc‐LuH_3_

**Figure 5:**
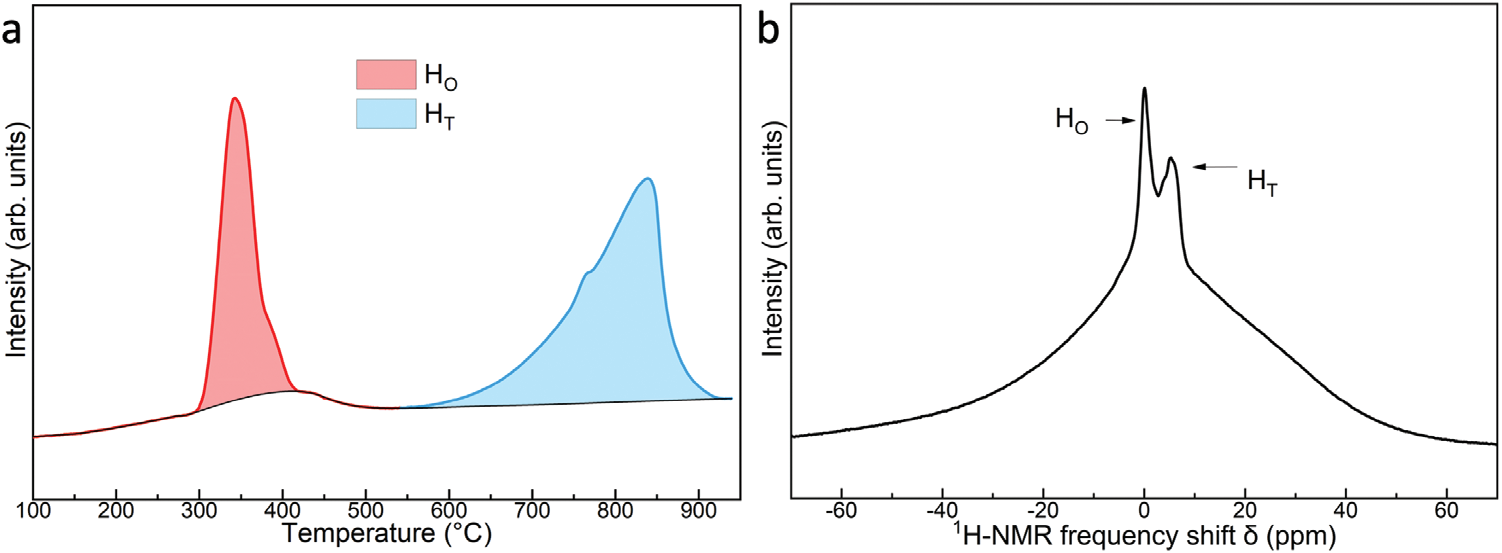
Determination of hydrogen positions in fcc‐LuH_3_. a) Thermal desorption profile for fcc‐LuH_3_ measured under He flow. b) ^1^H‐NMR spectrum of fcc‐LuH_3_.

To quantify the hydrogen content in the synthesized fcc‐LuH_3_, we utilized both temperature‐programmed desorption (TPD) and Nuclear Magnetic Resonance (NMR) measurements. These techniques provide semi‐quantitative evaluations of the hydrogen content. To investigate the thermal desorption behavior of the sample, a TPD experiment was conducted under a helium gas flow. Two prominent peaks were clearly observed in the temperature range of approximately 300–440 and 550–950 °C, respectively, which indicates hydrogen release with temperature increasing (**Figure** **5**a). The area ratio of low and high‐temperature peaks was ≈1:2, indicating a correlation with H_O_ and H_T_ sites, respectively. Notably, H_O_ atoms exhibited lower stability, evidenced by their release at lower temperatures compared to H_T_ atoms. The ^1^H‐NMR spectroscopy served as a tool for the investigation and analysis of hydrogen occupancy. The ^1^H‐NMR spectrum of fcc‐LuH_3_, as illustrated in Figure 5b, revealed distinct chemical shifts at +0.1 and +5.4 ppm. The two H chemical shifts suggest an association with H_O_ and H_T_ sites, respectively, aligning consistently with the outcomes of TPD results. The observed broad and extensive peak band is attributed to the presence of a paramagnetic Lu ion. Consequently, the integration of the peaks to determine the area and subsequently estimate the ratio of the two hydrogen atom sites becomes challenging. Based on the comprehensive analysis involving TPD and NMR, we have deduced that the fcc Lu hydride obtained under high‐temperature and high‐pressure synthesis corresponds to fcc‐LuH_3_.

### 2.9 The Absence of Superconductivity in fcc‐LuH_3_ at Ambient Conditions

**Figure 6:**
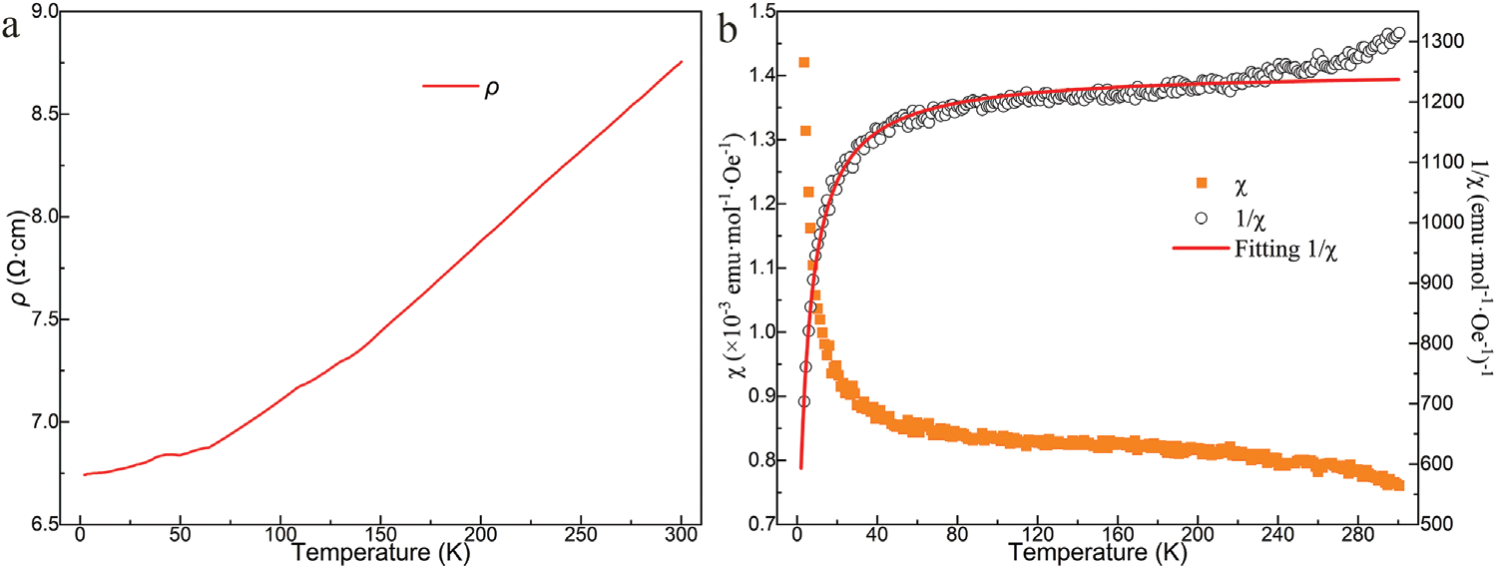
Low temperature electrical and magnetic measurement of fcc‐LuH_3_. a) Electrical resistivity measurement in the temperature range of 2 to 300 K. b) The magnetic susceptibility (left axis, filled symbols) and inverse magnetic susceptibility (right axis, open symbols) normalized per mole for fcc‐LuH_3_.

The non‐superconductivity of fcc‐LuH_3_ at ambient conditions was confirmed through low‐temperature electrical and magnetic measurements. Following the successful synthesis of the polycrystalline sample, its electrical and magnetic properties were analyzed using a Physical Property Measurement System (PPMS). **Figure** **6**a illustrates the temperature‐dependent resistivity (*ρ*‐*T*) of fcc‐LuH_3_ over a temperature range of 2 to 300 K. Notably, the resistivity decreases with decreasing temperature, indicating a classic metallic conductivity. The metallic behavior is also confirmed by DFT calculations (Figure S11, Supporting Information). Analysis of the electronic band structure reveals the metallic character of fcc‐LuH_3_, attributed to the overlap of conduction and valence bands at the Fermi level. Examination of the partial electronic density of states indicates that Lu primarily contributes to the density of states at the Fermi level. Figure 6b presents the temperature‐dependent magnetic susceptibility of the sample under a magnetic field of 1000 Oe, over a temperature range from 2 to 300 K. The magnetization–temperature (*M*–*T*) curve displays paramagnetic behavior. Given that the curvature of the inverse susceptibility χ^−1^ versus T is positive, indicating a positive χ_0_, a modified version of the Curie‐Weiss law is employed to fit the χ^−1^ − *T* curve. The modified Curie–Weiss equation is

(1) $$\chi =\frac{C}{T-\theta_{\mathrm{cw}}}+\chi_{0}$$

(2) $$\chi^{-1}=\frac{T-\theta_{\mathrm{cw}}}{\chi_{0}\cdot \left(T-\theta_{\mathrm{cw}}\right)+C}$$

yielding Curie constant *C* = 2.92(6) × 10^−3^ emu K mol^−1^, Curie‐Weiss temperature θ_cw_ = −1.3(1) K, and temperature‐independent susceptibility χ_0_ = 8 × 10^−4^ emu mol^−1^. Given the metallic and paramagnetic properties of fcc‐LuH_3_ observed through low‐temperature electrical and magnetic measurements, no further superconductivity measurements were conducted. The sample was retrieved from low temperature, and the morphology of the sample remains unchanged. The subsequent XRD pattern confirms its stability after cooling to 2 K, exhibiting the typical diffraction pattern of fcc‐LuH_3_ (Figure S12, Supporting Information).

## 3 Conclusion

In conclusion, we have successfully synthesized bulk lutetium hydrides of LuH_2_, hcp‐LuH_3_, and fcc‐LuH_3_, and constructed a synthesis phase diagram. The high‐pressure phase fcc‐LuH_3_ is retained at ambient conditions through a high‐temperature and high‐pressure method using LVP. DFT calculations of lattice dynamics with anharmonic and temperature effects confirm that fcc‐LuH_3_ is stable at ambient conditions. The hydrogen content of fcc‐LuH_3_ is confirmed through the TPD and NMR methods. Low‐temperature electrical and magnetic measurements reveal the absence of superconductivity in fcc‐LuH_3_ under ambient conditions. This study establishes a comprehensive synthesis strategy for lutetium hydrides, and this approach enhances our understanding of the high‐temperature and high‐pressure method used in hydrides with superconductivity.

## Conflict of Interest

The authors declare no conflict of interest.

## Supporting information

Supporting Information

## Data Availability

The data that support the findings of this study are available from the corresponding author upon reasonable request.

## References

[1] H. Wang , J. S. Tse , K. Tanaka , T. Iitaka , Y. Ma , Proc. Natl. Acad. Sci. USA 2012, 109, 6463. 22492976 10.1073/pnas.1118168109 PMC3340045

[2] D. F. Duan , Y. X. Liu , F. B. Tian , D. Li , X. L. Huang , Z. L. Zhao , H. Y. Yu , B. B. Liu , W. J. Tian , T. Cui , Sci. Rep. 2014, 4, 6.

[3] L. Ma , K. Wang , Y. Xie , X. Yang , Y. Wang , M. Zhou , H. Liu , X. Yu , Y. Zhao , H. Wang , G. Liu , Y. Ma , Phys. Rev. Lett. 2022, 128, 167001. 35522494 10.1103/PhysRevLett.128.167001

[4] F. Peng , Y. Sun , C. J. Pickard , R. J. Needs , Q. Wu , Y. M. Ma , Phys. Rev. Lett. 2017, 119, 6. 10.1103/PhysRevLett.119.107001 28949166

[5] Z. M. Geballe , H. Y. Liu , A. K. Mishra , M. Ahart , M. Somayazulu , Y. Meng , M. Baldini , R. J. Hemley , Angew. Chem., Int. Ed. 2018, 57, 688. 10.1002/anie.201709970 29193506

[6] X. Li , X. Huang , D. Duan , C. J. Pickard , D. Zhou , H. Xie , Q. Zhuang , Y. Huang , Q. Zhou , B. Liu , T. Cui , Nat. Commun. 2019, 10, 3461. 31371729 10.1038/s41467-019-11330-6 PMC6671988

[7] D. V. Semenok , A. G. Kvashnin , A. G. Ivanova , V. Svitlyk , V. Y. Fominski , A. V. Sadakov , O. A. Sobolevskiy , V. M. Pudalov , I. A. Troyan , A. R. Oganov , Mater. Today 2020, 33, 36.

[8] P. Kong , V. S. Minkov , M. A. Kuzovnikov , A. P. Drozdov , S. P. Besedin , S. Mozaffari , L. Balicas , F. F. Balakirev , V. B. Prakapenka , S. Chariton , D. A. Knyazev , E. Greenberg , M. I. Eremets , Nat. Commun. 2021, 12, 5075. 34417471 10.1038/s41467-021-25372-2 PMC8379216

[9] A. P. Drozdov , M. I. Eremets , I. A. Troyan , V. Ksenofontov , S. I. Shylin , Nature 2015, 525, 73. 26280333 10.1038/nature14964

[10] A. P. Drozdov , P. P. Kong , V. S. Minkov , S. P. Besedin , M. A. Kuzovnikov , S. Mozaffari , L. Balicas , F. F. Balakirev , D. E. Graf , V. B. Prakapenka , Nature 2019, 569, 528. 31118520 10.1038/s41586-019-1201-8

[11] N. Dasenbrock‐Gammon , E. Snider , R. McBride , H. Pasan , D. Durkee , N. Khalvashi‐Sutter , S. Munasinghe , S. E. Dissanayake , K. V. Lawler , A. Salamat , R. P. Dias , Nature 2023, 615, 244. 36890373 10.1038/s41586-023-05742-0

[12] X. Ming , Y.‐J. Zhang , X. Zhu , Q. Li , C. He , Y. Liu , T. Huang , G. Liu , B. Zheng , H. Yang , J. Sun , X. Xi , H.‐H. Wen , Nature 2023, 620, 72. 37168015 10.1038/s41586-023-06162-w PMC10396964

[13] X. Xing , C. Wang , L. Yu , J. Xu , C. Zhang , M. Zhang , S. Huang , X. Zhang , Y. Liu , B. Yang , X. Chen , Y. Zhang , J. Guo , Z. Shi , Y. Ma , C. Chen , X. Liu , Nat. Commun. 2023, 14, 5991. 37752133 10.1038/s41467-023-41777-7 PMC10522599

[14] Y. Sun , F. Zhang , S. Wu , V. Antropov , K.‐M. Ho , Phys. Rev. B 2023, 108, L020101.

[15] Z. Huo , D. Duan , T. Ma , Z. Zhang , Q. Jiang , D. An , H. Song , F. Tian , T. Cui , Matter Radiat. Extremes 2023, 8, 038402.

[16] F. Xie , T. Lu , Z. Yu , Y. Wang , Z. Wang , S. Meng , M. Liu , Chinese Phys. Lett. 2023, 40, 057401.

[17] X. Tao , A. Yang , S. Yang , Y. Quan , P. Zhang , Sci. Bull. 2023, 68, 1372. 10.1016/j.scib.2023.06.007 37349163

[18] K. P. Hilleke , X. Wang , D. Luo , N. Geng , B. Wang , F. Belli , E. Zurek , Phys. Rev. B 2023, 108, 014511.

[19] P. P. Ferreira , L. J. Conway , A. Cucciari , S. Di Cataldo , F. Giannessi , E. Kogler , L. T. F. Eleno , C. J. Pickard , C. Heil , L. Boeri , Nat. Commun. 2023, 14, 5367. 37666834 10.1038/s41467-023-41005-2 PMC10477194

[20] Đ. Dangić , P. Garcia‐Goiricelaya , Y.‐W. Fang , J. Ibañez‐Azpiroz , I. Errea , Phys. Rev. B 2023, 108, 064517.

[21] Y.‐J. Zhang , X. Ming , Q. Li , X. Zhu , B. Zheng , Y. Liu , C. He , H. Yang , H.‐H. Wen , Y.‐J. Zhang , X. Ming , Q. Li , X. Zhu , B. Zheng , Y. Liu , C. He , H. Yang , H.‐H. Wen , Sci China Phys Mech Astron 2023, 66, 287411.

[22] S. Zhang , J. Bi , R. Zhang , P. Li , F. Qi , Z. Wei , Y. Cao , AIP Adv. 2023, 13, 065117.

[23] N. Wang , J. Hou , Z. Liu , T. Lu , P. Shan , C. Chai , S. Jin , L. Ma , L. Shi , X. Wang , Y. Long , Y. Liu , H. Zhang , X. Dong , S. Meng , M. Liu , J. Cheng , Sci China Phys Mech Astron 2023, 66, 297412.

[24] C. Tresca , P. M. Forcella , A. Angeletti , L. Ranalli , C. Franchini , M. Reticcioli , G. Profeta , arXiv.2308.03619 2023. 10.1038/s41467-024-51348-z PMC11343858 39179540

[25] A. Sufyan , J. A. Larsson , ACS Omega 2023, 8, 9607. 36936326 10.1021/acsomega.3c00207 PMC10018709

[26] P. Shan , N. Wang , X. Zheng , Q. Qiu , Y. Peng , J. Cheng , Chinese Phys. Lett. 2023, 40, 046101.

[27] N. P. Salke , A. C. Mark , M. Ahart , R. J. Hemley , arXiv.2306.06301 2023.

[28] D. Peng , Q. Zeng , F. Lan , Z. Xing , Y. Ding , H. Mao , Matter Radiat. Extremes 2023, 8, 058401.

[29] Z. Ouyang , M. Gao , Z.‐Y. Lu , arXiv.2306.13981 2023.

[30] O. Moulding , S. Gallego‐Parra , Y. Gao , P. Toulemonde , G. Garbarino , P. De Rango , S. Pairis , P. Giroux , M.‐A. Méasson , Phys. Rev. B 2023, 108, 214505.

[31] R. Lv , W. Tu , D. Shao , Y. Sun , W. Lu , Chinese Phys. Lett. 2023, 40, 117401.

[32] Z. Li , X. He , C. Zhang , K. Lu , B. Min , J. Zhang , S. Zhang , J. Zhao , L. Shi , Y. Peng , S. Feng , Z. Deng , J. Song , Q. Liu , X. Wang , R. Yu , L. Wang , Y. Li , J. D. Bass , V. Prakapenka , S. Chariton , H. Liu , C. Jin , Sci China Phys Mech Astron 2023, 66, 267411.

[33] S.‐W. Kim , L. J. Conway , C. J. Pickard , G. L. Pascut , B. Monserrat , Nat. Commun. 2023, 14, 7360. 37963870 10.1038/s41467-023-42983-z PMC10646004

[34] Q. Jiang , D. Duan , H. Song , Z. Zhang , Z. Huo , T. Cui , Y. Yao , arXiv.2302.02621 2023.

[35] J. Guo , S. Cai , D. Wang , H. Shu , L. Yang , P. Wang , W. Wang , H. Tian , H. Yang , Y. Zhou , J. Zhao , J. Han , J. Li , Q. Wu , Y. Ding , W. Yang , T. Xiang , H. Mao , L. Sun , Chinese Phys. Lett. 2023, 40, 097401.

[36] J. Du , W. Sun , X. Li , F. Peng , Phys. Chem. Chem. Phys. 2023, 25, 13320. 37133917 10.1039/d3cp00604b

[37] S. Cai , J. Guo , H. Shu , L. Yang , P. Wang , Y. Zhou , J. Zhao , J. Han , Q. Wu , W. Yang , T. Xiang , H. Mao , L. Sun , Matter Radiat. Extremes 2023, 8, 048001.

[38] N. Dasenbrock‐Gammon , E. Snider , R. McBride , H. Pasan , D. Durkee , N. Khalvashi‐Sutter , S. Munasinghe , S. E. Dissanayake , K. V. Lawler , A. Salamat , R. P. Dias , Nature 2023, 624, 460. 37935926 10.1038/s41586-023-06774-2

[39] A. Pebler , W. E. Wallace , J. Phys. Chem. 1962, 66, 148.

[40] T. Palasyuk , M. Tkacz , Solid State Commun. 2005, 133, 481.

[41] T. Dierkes , J. Plewa , T. Jüstel , J. Alloys Compd. 2017, 693, 291.

[42] M. Shao , S. Chen , W. Chen , K. Zhang , X. Huang , T. Cui , Inorg. Chem. 2021, 60, 15330. 34590849 10.1021/acs.inorgchem.1c01960

[43] R. Kataoka , N. Taguchi , M. Kitta , N. Takeichi , R. Utsumi , H. Saitoh , M. Nozaki , A. Kamegawa , Mater. Today Commun. 2022, 31, 103265.

[44] R. Kataoka , T. Kimura , N. Takeichi , A. Kamegawa , Inorg. Chem. 2018, 57, 4686. 29620366 10.1021/acs.inorgchem.8b00409

[45] R. Lucrezi , P. P. Ferreira , M. Aichhorn , C. Heil , Nat. Commun. 2024, 15, 441. 38199988 10.1038/s41467-023-44326-4 PMC10781996

[46] W. A. Grosshans , W. B. Holzapfel , Phys. Rev. B 1992, 45, 5171. 10.1103/physrevb.45.5171 10000231

[47] C.‐M. Lin , K.‐T. Wu , T.‐L. Hung , H.‐S. Sheu , M.‐H. Tsai , J.‐F. Lee , J.‐J. Lee , Solid State Commun. 2010, 150, 1564.

[48] S. Jiang , J. Liu , C. Lin , L. Bai , W. Xiao , Y. Zhang , D. Zhang , X. Li , Y. Li , L. Tang , J. Appl. Phys. 2010, 108, 083541.

[49] X. Hao , X. Wei , H. Liu , X. Song , R. Sun , G. Gao , Y. Tian , Phys. Rev. Research 2023, 5, 043238.

