# Supplementary material for: Stabilization of High‐Pressure Phase of Face‐Centered Cubic Lutetium Trihydride at Ambient Conditions
Source: Adv Sci (Weinh). 2024 May 22;11(29):2401642. doi: 10.1002/advs.202401642 (PMC11304239; doi:10.1002/advs.202401642)
Supplement: Supplementary file 1 — Supporting Information [file ADVS-11-2401642-s001.docx]

**Supporting Information**

**Of**

**Absence of Superconductivity in Face-centered Cubic Lutetium Trihydride at Ambient Conditions**

Xin Li^+^,* Ying Wang^+^, Yuhao Fu, Simon A.T. Redfern, Shuqing Jiang, Pinwen Zhu, and Tian Cui*

[*] Dr. Xin Li, Dr. Ying Wang, Prof. Yuhao Fu, Prof. Shuqing Jiang, Prof Pinwen Zhu
Synergetic Extreme Condition High-Pressure Science Center, State Key Laboratory of Superhard Materials, College of Physics,
Jilin University
Changchun 130012, China
E-mail: xin_li@jlu.edu.cn

Dr. Xin Li, Prof. Simon A.T. Redfern
Asian School of the Environment
Nanyang Technological University
50 Nanyang Avenue, 639798

Prof Tian Cui
Institute of High Pressure Physics, School of Physical Scientific and Technology
Ningbo University
Ningbo 315211, China
E-mail: cuitian@nbu.edu.cn

[^+^] These authors contributed equally to this work.

**Methods**

**High-temperature and high-pressure synthesis experiments in large volume press.** The starting materials used were Lu pieces from Hebei Rechen New Material Technology Co., Ltd., and LuH_2_ powder from Adamas and Aladdin. The Lu pieces, characterized by a silver color, exhibited a high-purity hcp structure, as confirmed by XRD analysis (Figure S11). In contrast, the dark-blue-colored LuH_2_ powders displayed a high-purity fcc structure according to their XRD pattern (Figure S12). Ammonia borane (BH_3_NH_3_, Adamas, 98%+) served as the hydrogen source, consistent with previous reports.^[1–3]^ NH_4_Cl (Adamas,99.9%) and CaH_2_ (Adamas 97%+) were mixed in a molar ratio of 2:8 to serve as the nitrogen and hydrogen source.^[4,5]^ The sample preparation for high-pressure experiments was conducted in an Ar-filled glove box with low concentrations of H_2_O (<1 ppm) and O_2_ (<1 ppm). Lu or LuH_2_ samples were sandwiched in an excess amount of BH_3_NH_3_ or mixture of NH_4_Cl and CaH_2_. These components were compressed into cylindrical pellets with 4 mm in diameter and 4 mm in height, then sealed within hexagonal boron nitride (h*-*BN) capsules. Subsequently, the sealed h*-*BN capsules were placed in a graphite cylinder heater and subjected to high pressure and high temperature conditions using a cubic type of multi-anvils apparatus (SPD 6×600 T). The pressure calibration of the oil loading curve is established through the identification of phase transitions occurring at 2.55 GPa for Bi and at 5.5 GPa for Ba. After each experiment, the capsules were carefully removed, and sintered specimens were mechanically cleaned in an Ar-filled glove box for further analysis. Specific details regarding the experimental conditions for each individual experiment are discussed in the results and discussions section.

**Structure characterization.** The structural properties of the samples were characterized using a Rigaku D/max-2005 powder X-ray diffractometer with Cu Kα (λ = 1.5406 Å) radiation and a Rigaku R-AXIS-RAPID Ⅱ X-ray diffractometer with Cu Kα (λ = 1.5406 Å) radiation. The X-ray diffraction (XRD) patterns were indexed and refined using both the Reflex module of the Materials Studio Program^[6]^ and GSAS II^[7]^. All the provided lattice parameters were determined under ambient conditions. Additionally, the microstructures of the synthesized sample were examined using scanning electron microscopy (SEM) with a FEI Magellan 400 instrument to investigate the morphology.

**Properties measurement.** The temperature programmed desorption (AutoChem 2920) under He gas flow was employed to measure the thermal desorption behavior of the samples. Resistance-temperature (R-T) and magnetization-temperature (M-T) were measured in the range of 2 K to 300 K using a commercial apparatus (Quantum Design Physical Properties Measurement System, PPMS).

**Computational details**

The Vienna Ab initio Simulation Package (VASP) was used to perform Density Functional Theory (DFT) calculations on structure relaxations, total energies, phonons, and dynamics^[8]^. The Perdew, Burke, and Ernzerhof (PBE-GGA)^[9]^ generalized gradient approximation was employed, and the projector-augmented wave (PAW)^[10,11]^ approach was used to describe the core electrons and their effects on valence orbitals. The valence electron configuration of 5*s*²5*p*⁶4*f*¹⁴5*d*¹6*s*² and 1*s*¹ was used for the Lu and H atoms, respectively. A plane-wave kinetic-energy cut-off of 1000 eV was used for hard PAW potentials, and dense Monkhorst–Pack k-points grids with a reciprocal space resolution of 2π × 0.03 Å^−1^ were employed to sample the Brillouin zone. The temperature-dependent effective potential (TDEP) method was used to extract the anharmonic effects and temperature effects^[12]^. The molecular dynamics simulations were performed using 128-atom (2 × 2 × 2) supercells of fcc-LuH₃ with a simulation time of 50 ps and a time step of 1 fs. The phonon dispersion relations of fcc-LuH_3_ were obtained from second-order interatomic force constants (IFCs) using the PHONOPY package^[13]^.

**
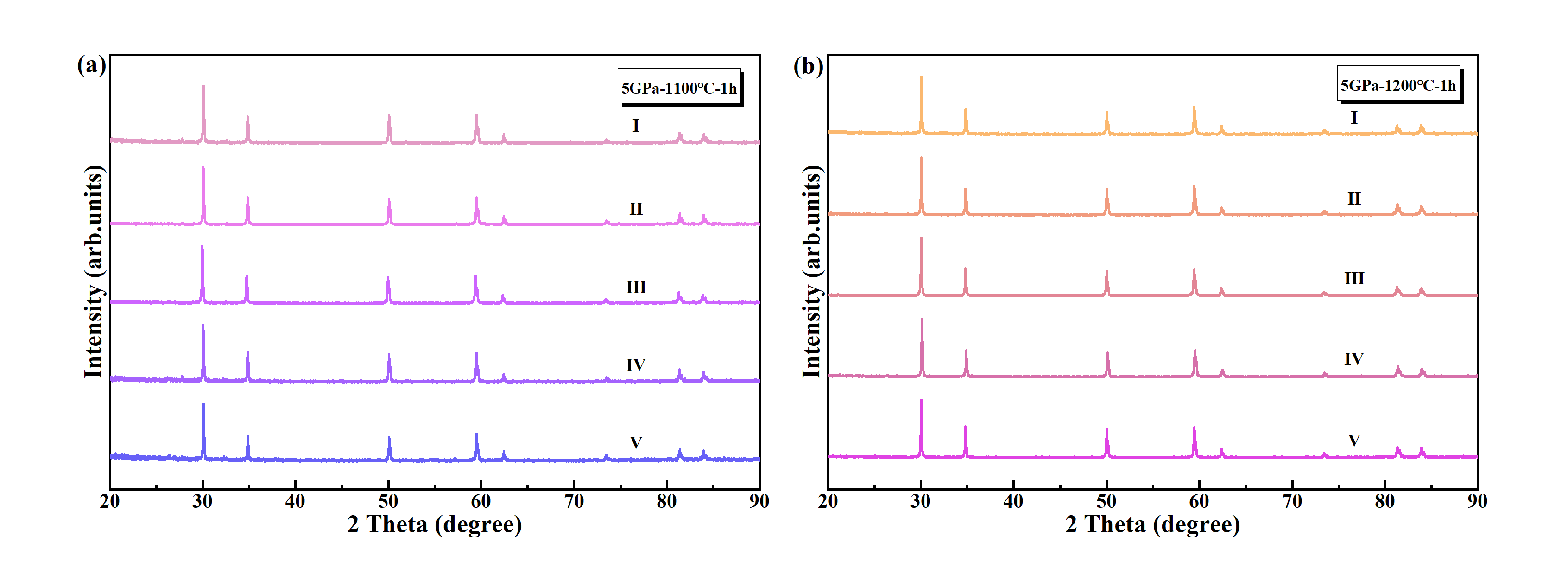
**

**Figure S1.** Powder XRD patterns of other ten fcc-LuH_3_ samples.


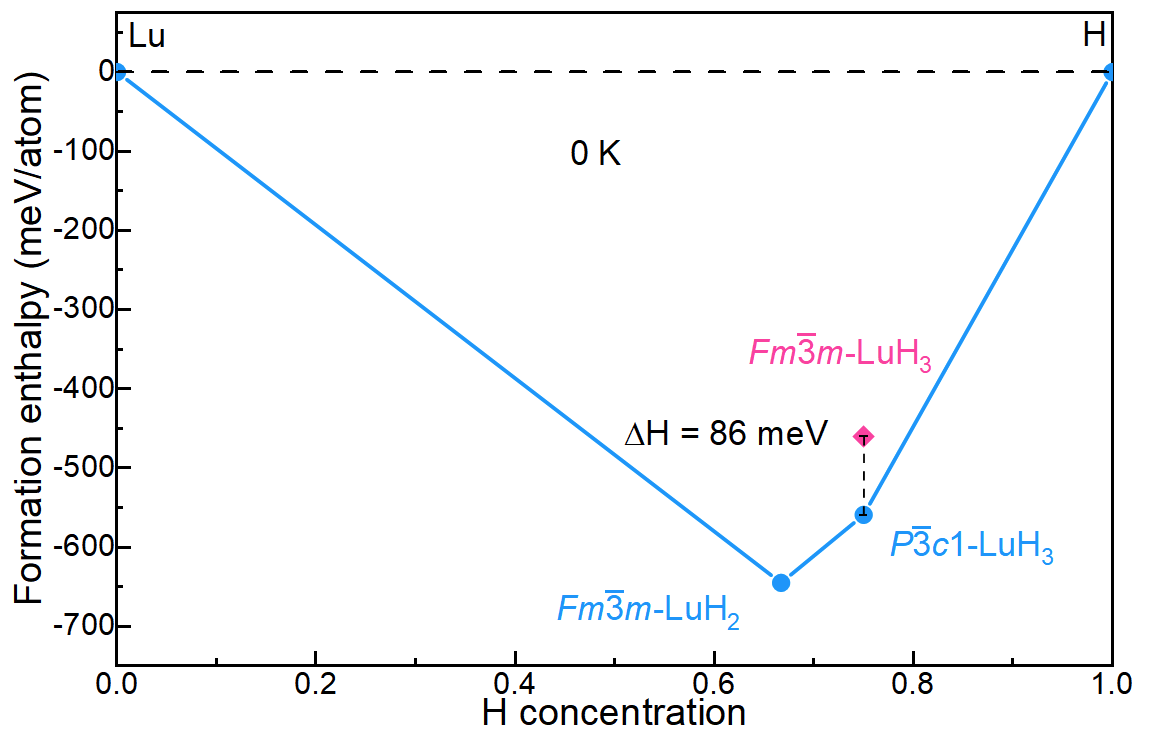


**Figure S2.** Convex hull of lutetium hydrides at 0 GPa.

**
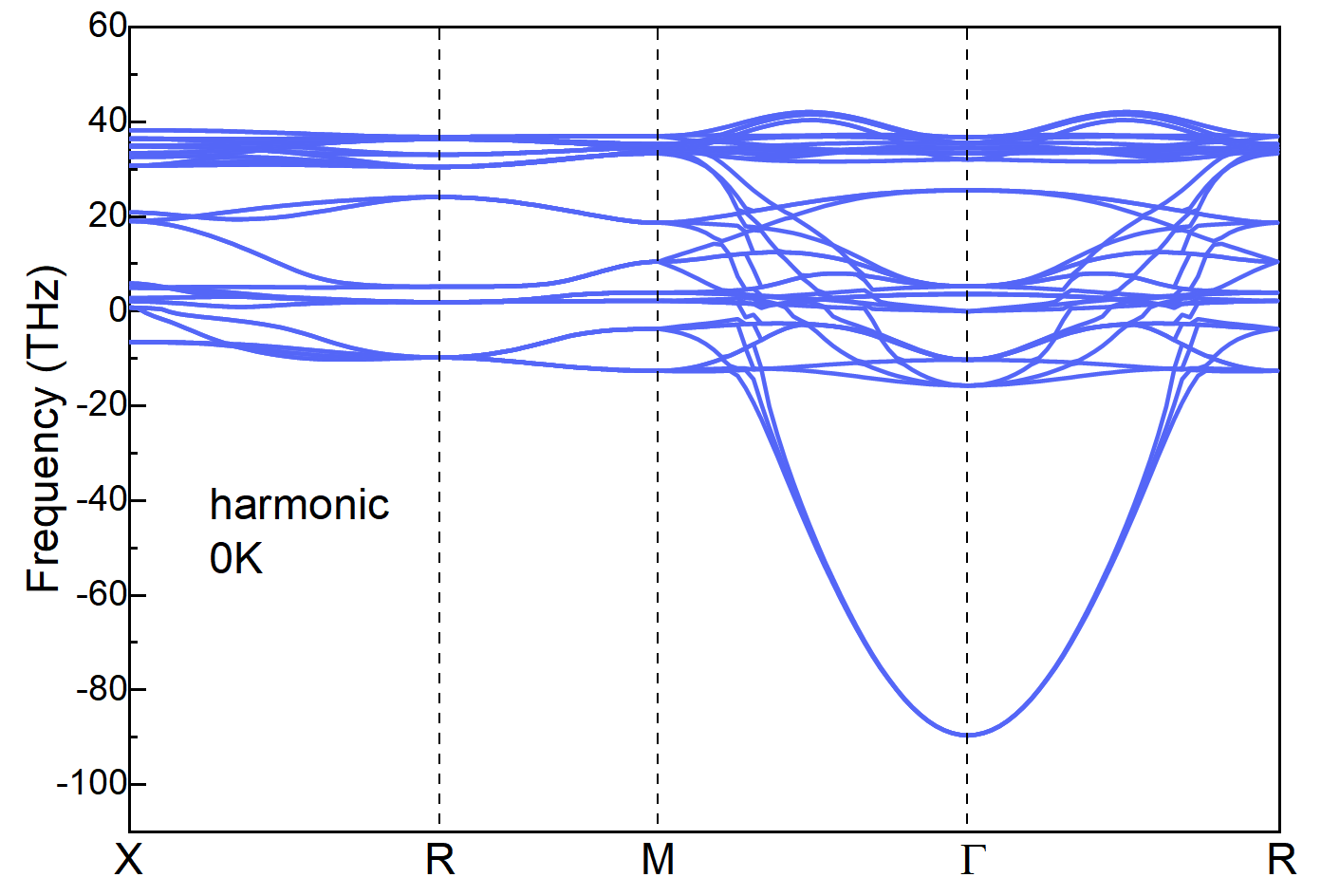
**

**Figure S3.** Harmonic phonon dispersions of fcc-LuH_3_ at 0 GPa and 0 K.


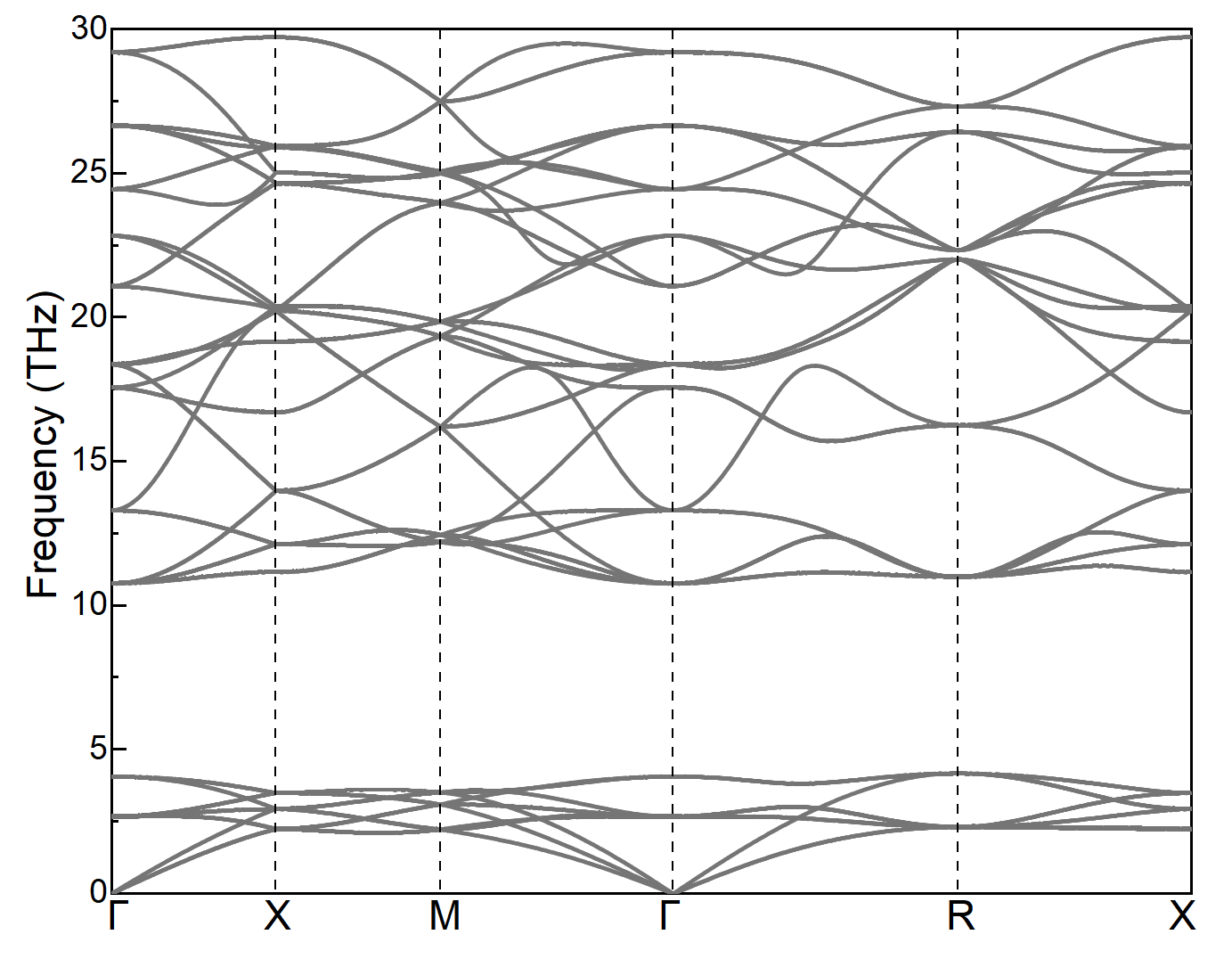


**Figure S4.** Anharmonic phonon dispersions of fcc-LuH_3_ at 0 GPa and 50 K.


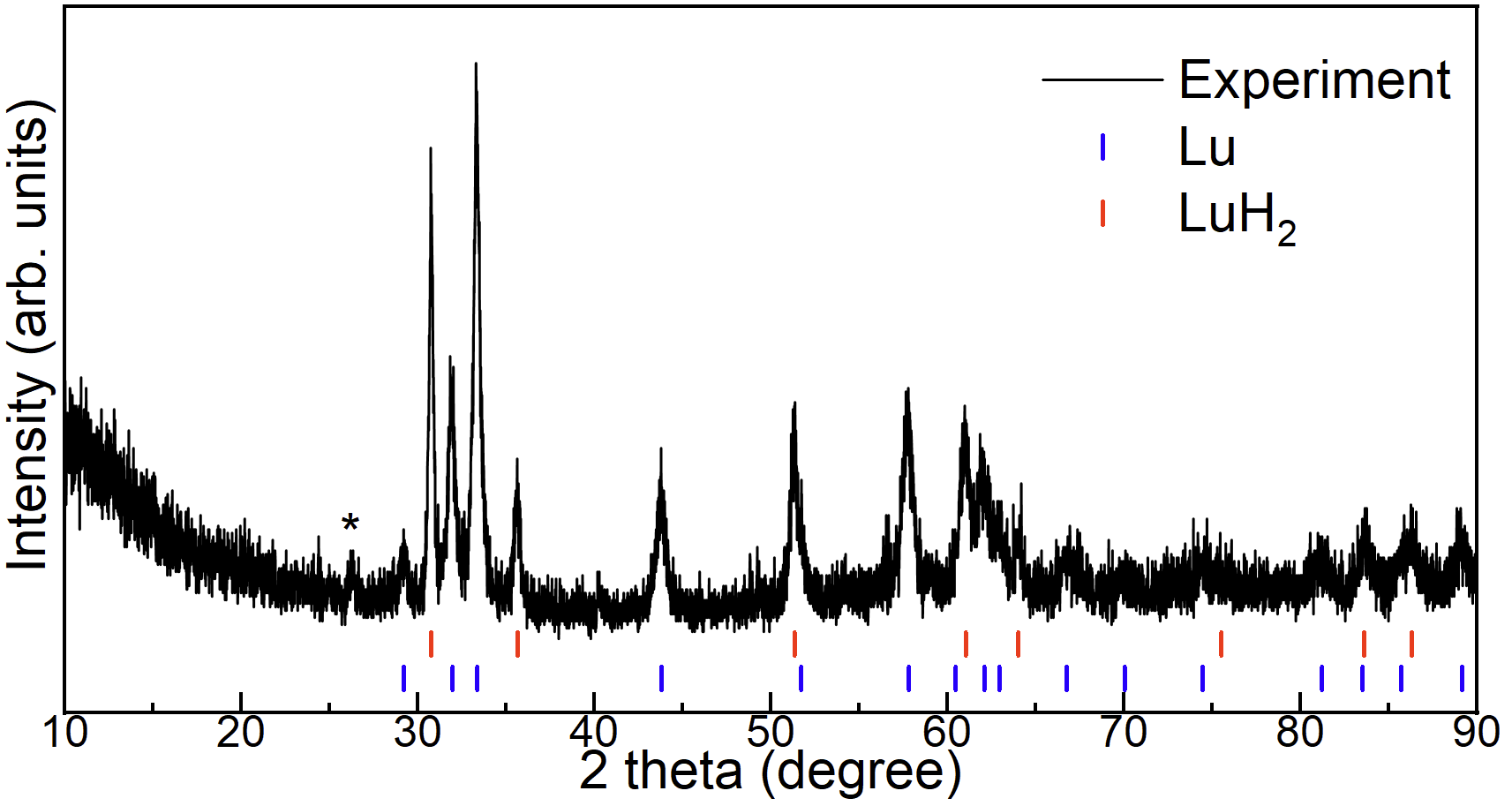


**Figure S5.** XRD pattern of synthesized LuH_2_. The positions of diffraction peaks for synthesized LuH_2_ and unreacted Lu are indicated by pink and gray ticks. The asterisk indicates the BN.


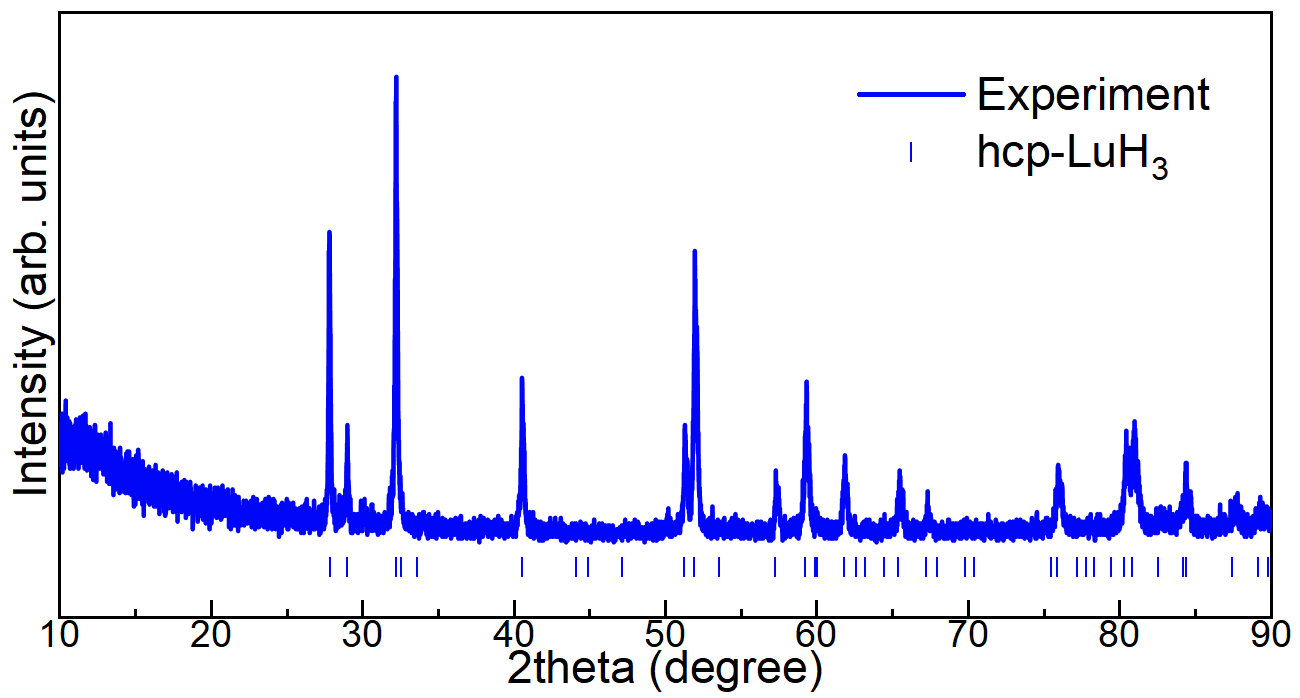


**Figure S6.** XRD pattern of synthesized hcp-LuH_3_.


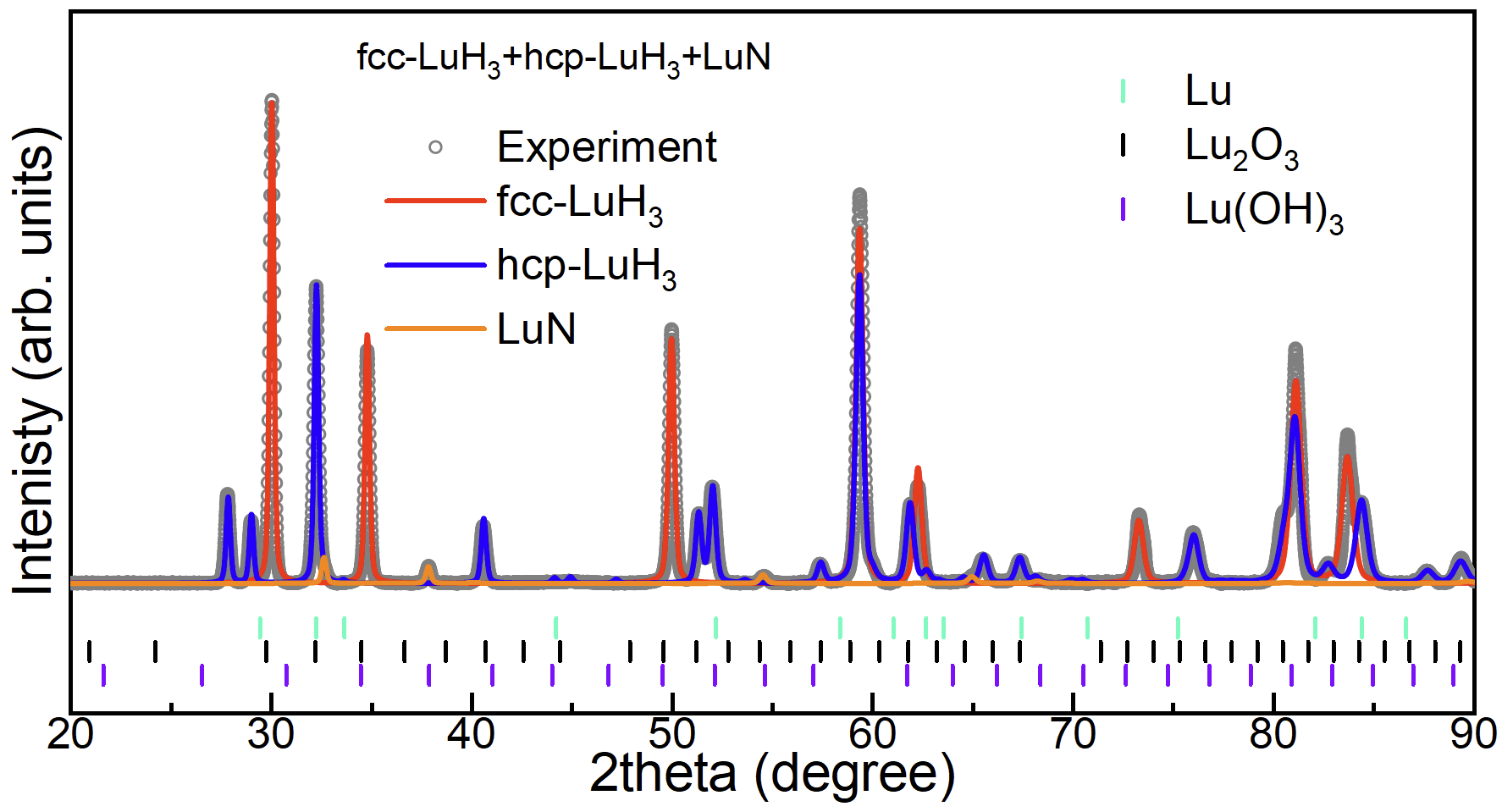


**Figure S7.** Indexing of sample and identification of impurities. The gray circles, pink line, blue line and orange line represent the experimental XRD pattern, simulated XRD of fcc-LuH_3_, simulated XRD of hcp-LuH_3_, and LuN, respectively. The expected positions of diffraction peaks for Lu, Lu_2_O_3_ and Lu(OH)_3_ are marked by pink, blue, orange, light blue, gray and purple ticks.


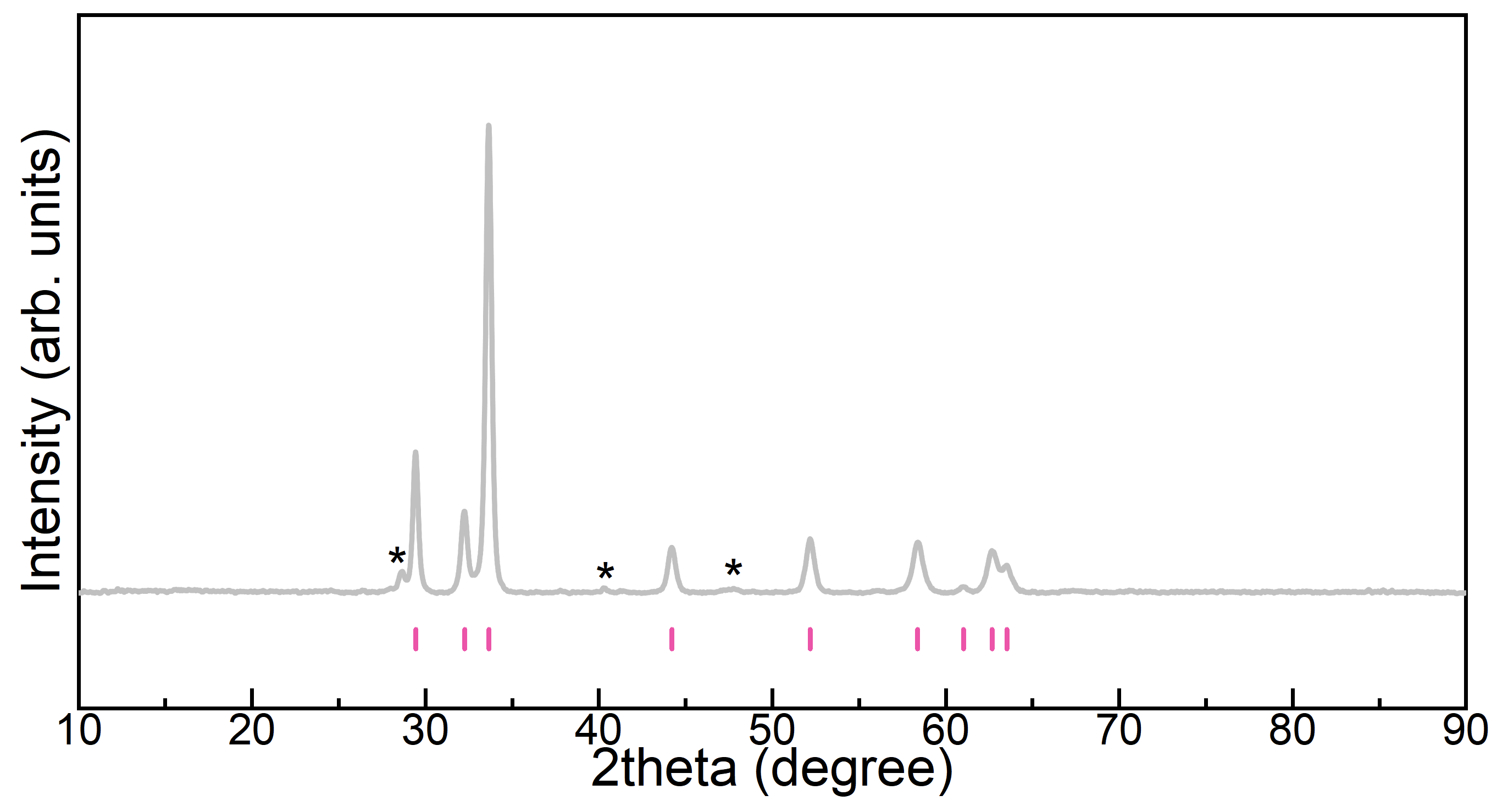


**Figure S8.** XRD pattern of staring material Lu. The asterisks indicate unknown impurities.


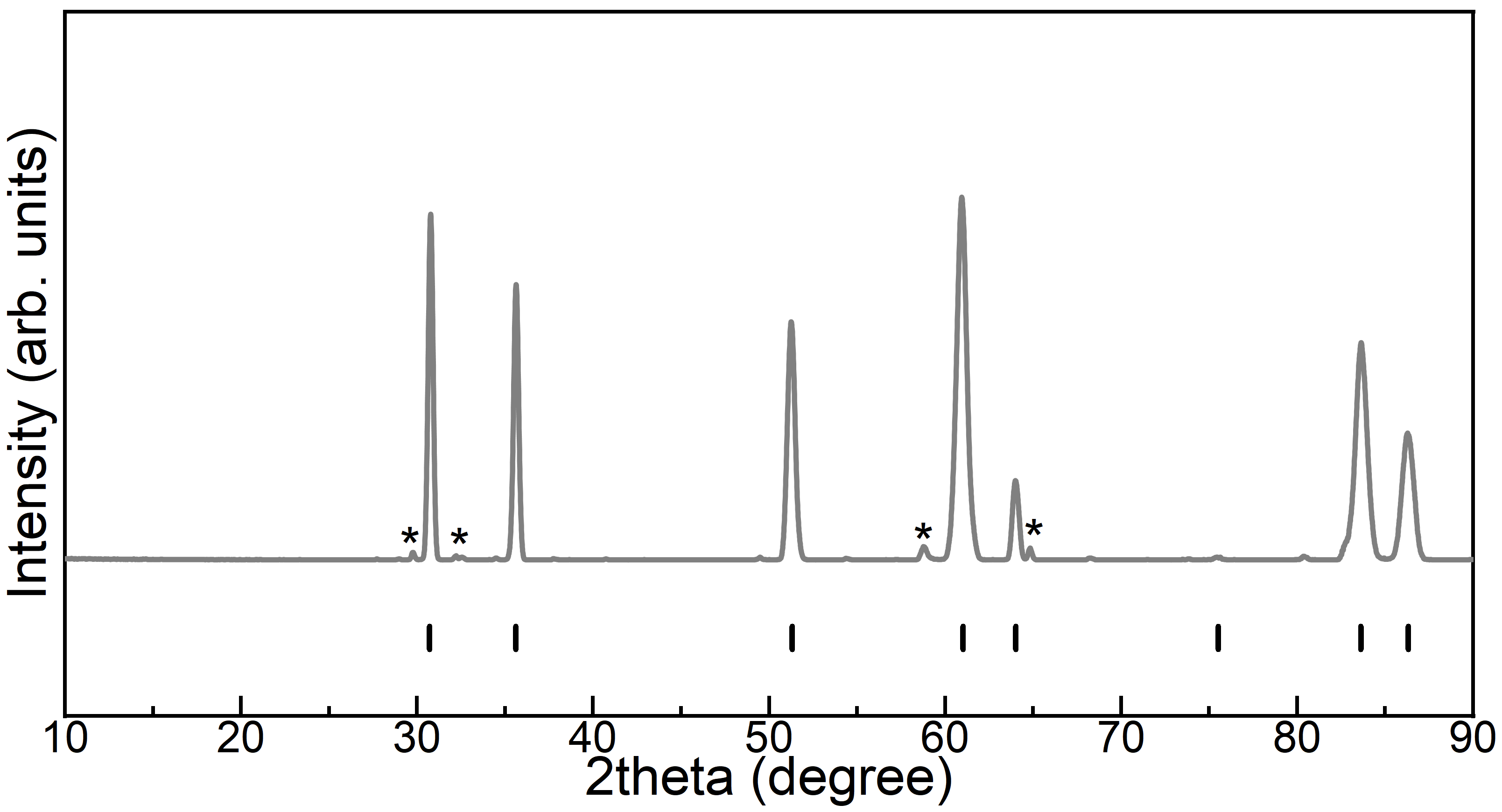


**Figure S9.** XRD pattern of starting material LuH_2._ The asterisks indicate unknown impurities.


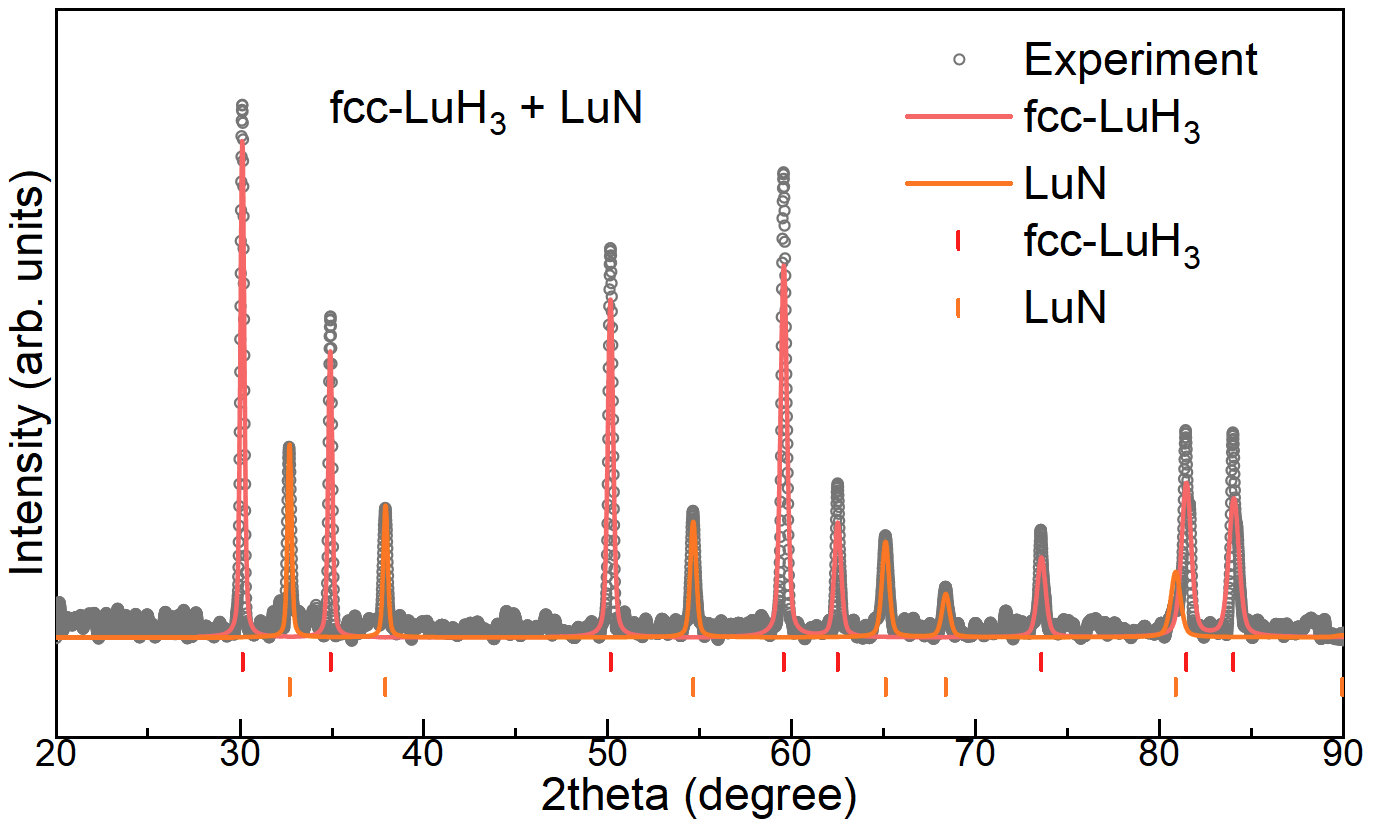


**Figure S10.** XRD pattern of fcc-LuH_3_ and LuN. A mass of LuN was detected when only the surface of the sample was cleaned.

**
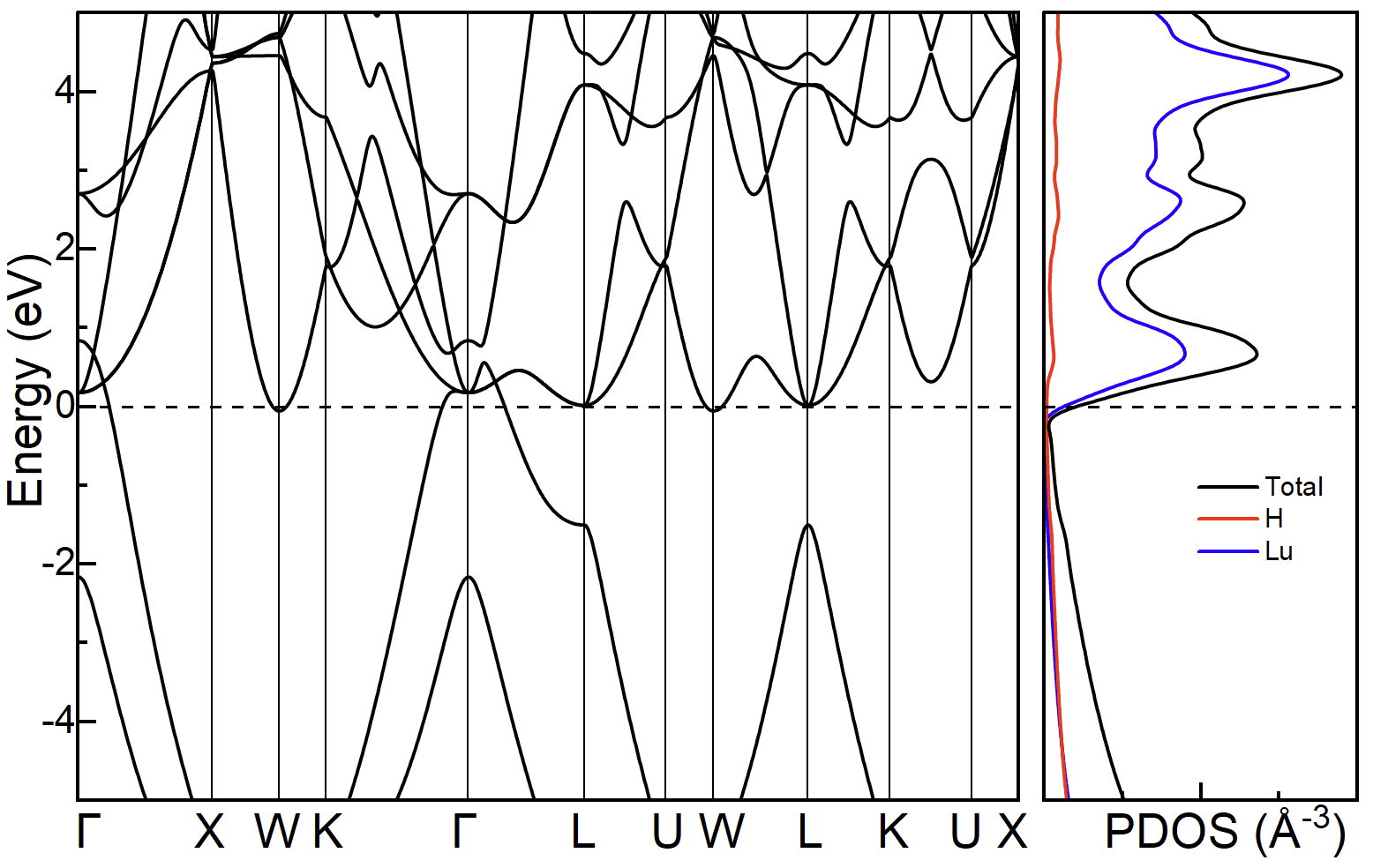
**

**Figure S11.** Calculated electronic band structure and partial density of electronic states of fcc-LuH_3_.


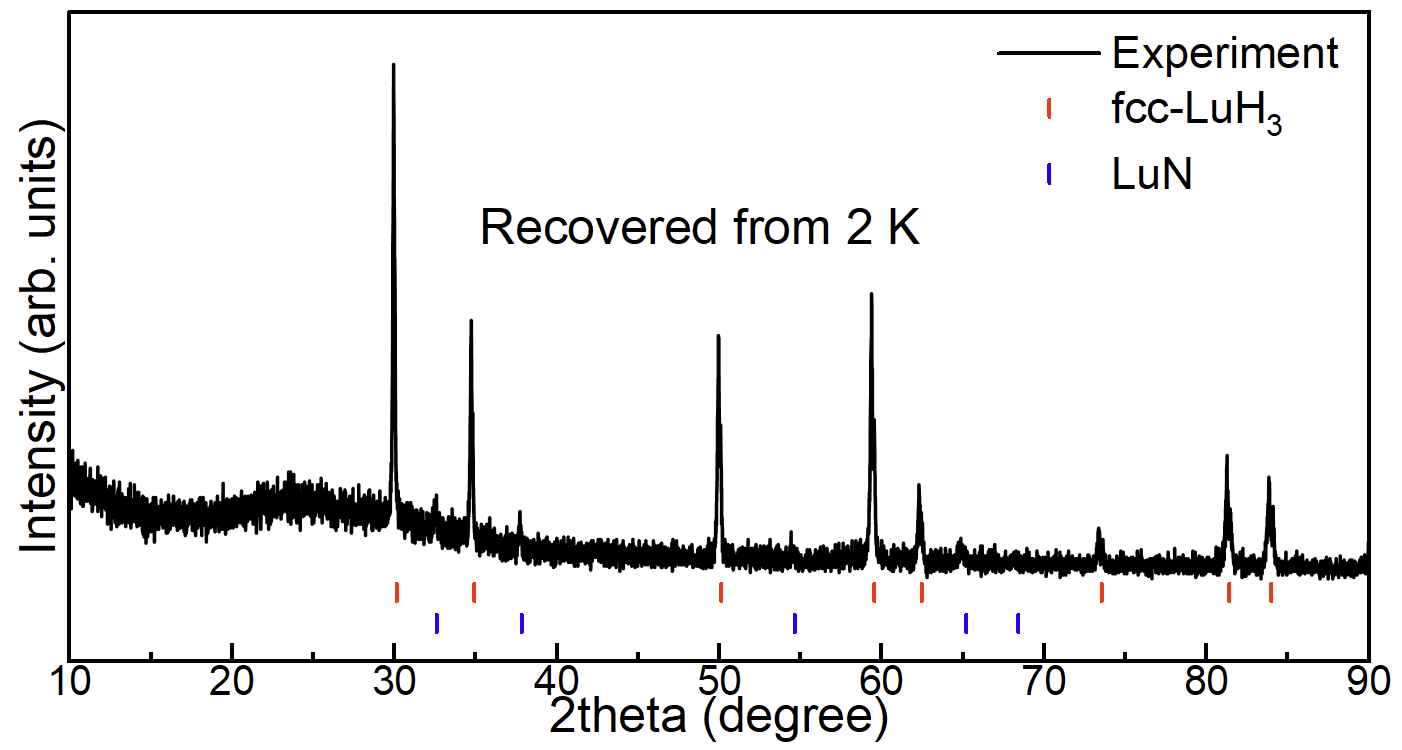


**Figure S12.** XRD pattern of fcc-LuH_3_ recovered from 2 K.

**
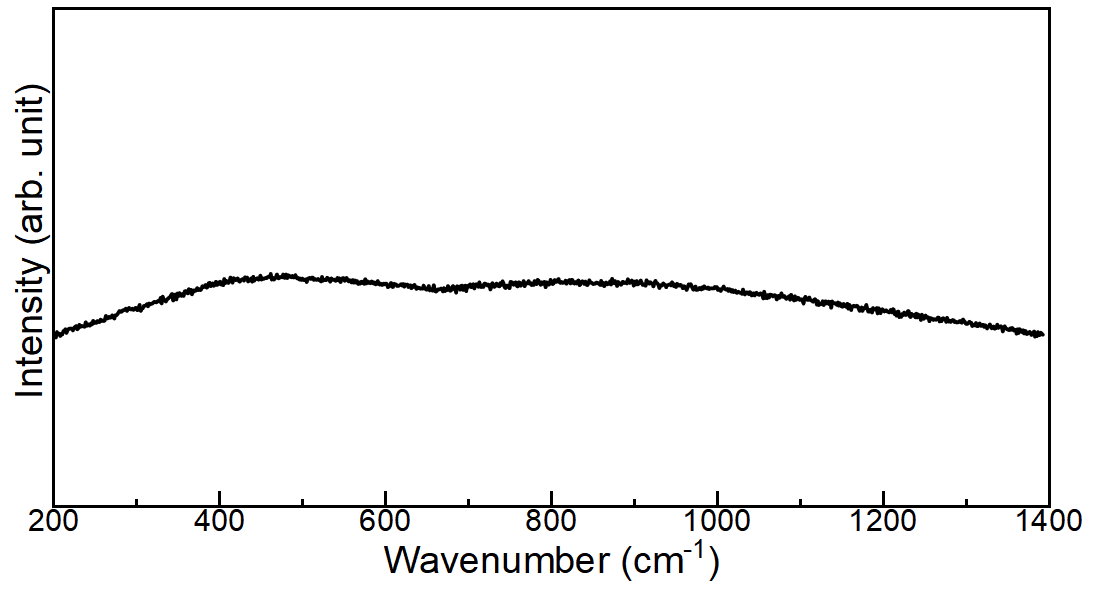
**

**Figure S13.** Raman spectra of fcc-LuH_3_ at ambient conditions.

**References**

[1] N. N. Wang, P. F. Shan, K. Y. Chen, J. P. Sun, P. T. Yang, X. L. Ma, B. S. Wang, X. H. Yu, S. Zhang, G. F. Chen, J.-G. Cheng, X. L. Dong, X. H. Chen, Z. X. Zhao, *Supercond. Sci. Technol.* **2021**, *34*, 034006.

[2] K. Spektor, W. A. Crichton, S. Filippov, S. I. Simak, A. Fischer, U. Häussermann, *Inorg. Chem.* **2020**, *59*, 16467–16473.

[3] K. Spektor, W. A. Crichton, S. Filippov, S. I. Simak, U. Häussermann, *Inorg. Chem.* **2019**, *58*, 11043–11050.

[4] X. Ming, Y.-J. Zhang, X. Zhu, Q. Li, C. He, Y. Liu, T. Huang, G. Liu, B. Zheng, H. Yang, J. Sun, X. Xi, H.-H. Wen, *Nature* **2023**, *620*, 72–77.

[5] X. Xing, C. Wang, L. Yu, J. Xu, C. Zhang, M. Zhang, S. Huang, X. Zhang, Y. Liu, B. Yang, X. Chen, Y. Zhang, J. Guo, Z. Shi, Y. Ma, C. Chen, X. Liu, *Nat. Commun.* **2023**, *14*, 5991.

[6] R. A. Young, *International union of crystallography* **1993**, *5*, 1–38.

[7] B. H. Toby, R. B. Von Dreele, *J Appl Crystallogr* **2013**, *46*, 544–549.

[8] G. Kresse, J. Furthmüller, *Phys. Rev. B* **1996**, *54*, 11169–11186.

[9] J. P. Perdew, K. Burke, M. Ernzerhof, *Phys. Rev. Lett.* **1996**, *77*, 3865–3868.

[10] G. Kresse, D. Joubert, *Phys. Rev. B* **1999**, *59*, 1758–1775.

[11] P. E. Blochl, *Phys. Rev. B* **1994**, *50*, 17953–17979.

[12] O. Hellman, P. Steneteg, I. A. Abrikosov, S. I. Simak, *Phys. Rev. B* **2013**, *87*, 104111.

[13] A. Togo, F. Oba, I. Tanaka, *Phys. Rev. B* **2008**, *78*, 9.
